# Role of filler and its heterostructure on moisture sorption mechanisms in polyimide films

**DOI:** 10.1038/s41598-018-35181-1

**Published:** 2018-11-15

**Authors:** Hom N. Sharma, Matthew P. Kroonblawd, Yunwei Sun, Elizabeth A. Glascoe

**Affiliations:** Lawrence Livermore National Laboratory, 7000 East Ave., Livermore, California, 94550 United States

## Abstract

Moisture sorption and diffusion exacerbate hygrothermal aging and can significantly alter the chemical and mechanical properties of polymeric-based components over time. In this study, we employ a multi-pronged multi-scale approach to model and understand moisture diffusion and sorption processes in polyimide polymers. A reactive transport model with triple-mode sorption (i.e., Henry’s, Langmuir, and pooling), experiments, and first principles atomistic computations were combined to synergistically explore representative systems of Kapton H and Kapton HN polymers. We find that the CaHPO_4_ processing aid used in Kapton HN increases the total moisture uptake (~0.5 wt%) relative to Kapton H. Henry’s mode is found to play a major role in moisture uptake for both materials, accounting for >90% contribution to total uptake.However, the pooling mode uptake in Kapton HN was ~5 times higher than in Kapton H. First principles thermodynamics calculations based on density functional theory predict that water molecules chemisorb (with binding energy  ~17–25 kcal/mol) on CaHPO_4_ crystal surfaces. We identify significant anisotropy in surface binding affinity, suggesting a possible route to tune and mitigate moisture uptake in Kapton-based systems through controlled crystal growth favoring exposure of CaHPO_4_ (101) surfaces during manufacturing.

## Introduction

Polyimide films are ubiquitous in electronics, circuit boards, and spacecrafts because of their thermal stability, chemical resistance, and mechanical properties^1–5^. Although these films are chemically compatible and extremely resilient to degradation, studies have demonstrated that they are hygroscopic and can uptake substantial amounts of moisture, relative to their weight, under normal atmospheric conditions^6–8^. One major concern is that over time, this latent moisture may outgas and diffuse into surrounding materials triggering hydrolytic degradation, metal corrosion, matrix cracking, micro-void generation, and/or interfacial delamination^2,9–11^. Any of these processes may lead to irreversible damage to the device. Understanding and predicting moisture uptake and outgassing in polyimides is important for establishing lifetimes and viability of electronics, circuit boards, spacecrafts, and many other devices and components that utilize these polymer films.

The hygrothermal properties of polyimides have been studied previously^7,8,12^, however, the production and availability of new polyimide formulations necessitates additional studies and enables the exploration of how small formulation changes can alter these properties. Kapton is a commercially available polyimide polymer manufactured by DuPont. It is synthesized using the monomers pyromellitic dianhydride (PMDA) and 4,4′-oxydianiline (ODA), and is reported to have large moisture uptake affinity (up to ~2.8 wt %)^6,7^. With a glass transition temperature (*T*_g_) of 360–410 °C, these materials are glassy polymers near ambient conditions. Two Kapton materials are considered here as representative systems, namely Kapton H and Kapton HN. Kapton HN contains the slip-additive calcium phosphate dibasic (CaHPO_4_) in addition to the base polymer in Kapton H^13^. These two materials were chosen to demonstrate the effects of fillers in moisture sorption and to quantify sorption modes using a multi-pronged approach including theory, experiments, and computations. Previous reports on Kapton films relied on equilibrium states and suggest that Henry’s sorption mode, dual-mode, and Fickian diffusion are the dominant processes, but provide limited mechanistic insights^7,14,15^. Modern gravimetric based dynamic vapor sorption (DVS) measurements, dynamic sorption/diffusion modeling, and atomistic modeling allow for a more complete investigation of the dynamics and mechanisms of these processes.

A polymeric material’s moisture sorption properties can be significantly altered by the addition of fillers, such as zeolites, silica, phosphate, or alumina compounds^16–18^. In many instances, fillers are intentionally added to alter these sorption properties. The filler may alter the polymeric material’s macro- and microstructure^19^, density, relaxation time, glass transition temperature (tan *δ*), specific heat, and may also have an affinity for moisture. For instance, the moisture uptake in a silica-filled polydimethylsiloxane (PDMS) polymer was found to be ~10 times higher than the unfilled PDMS sample^20^, whereas a marked decrease in the sorption of organic vapors was noted at low relative pressures in TiO_2_-filled polyvinylacetate^18^ and reduced moisture absorption on a polyimide 6 polymer due to cut glass fibers filler^21^. A deeper mechanistic and molecular-level understanding is required to unravel such complicated phenomena.

A traditional approach to investigate diffusion involves a Fickian-type (Case I) model (for example, the Crank and Park model)^22,23^, which simply relates the mass flux to the concentration gradient of each species. While useful, the solution from the Crank model is more suited to processes occurring on long time scales^23^. Non-Fickian diffusion is often treated by relating the total mass uptake *M* over time *t* as *M* ∝ *t*^*n*^, with pseudo-Fickian cases having *n* < 0.5 and anomalous cases with 0.5 < n < 1.0. Special cases are recovered with *n* = 0.5 (Fickian) and *n* = 1.0 (Case II sorption). Sorption kinetics, mostly implemented as equilibrium isotherms, have been explored using other models such as Langmuir, Freundlich, and Brunauer-Emmett-Teller^24,25^. In some cases, a dual-mode isotherm with Langmuir and Henry’s type sorption modes has been proposed^26–31^, which is reasonable for obtaining correlations, but lacks predictive capacity^32^.

The above treatments are limited to equilibrium states, which is not sufficient in practice for modeling realistic, dynamically changing systems involving multi-material and multi-gas (and vapor) interactions with unknown aging and compatibility scenarios. Moreover, some materials show unique sorption behavior at higher relative humidities, where water molecules start to aggregate leading to an elevated moisture uptake. This type of clustering mode (or pooling mode) is significantly different from Henry’s or Langmuir-type sorption modes and needs a different mathematical formulation beyond the dual-mode sorption model^20,32^. While the Zimm-Lundberg clustering model^33,34^ is commonly used to treat pooling behavior in equilibrium situations, it is not sufficient for transient processes. Furthermore, these macro- and meso-scale models lack molecular-level insights to explain material-water interactions.

**Figure 1:**
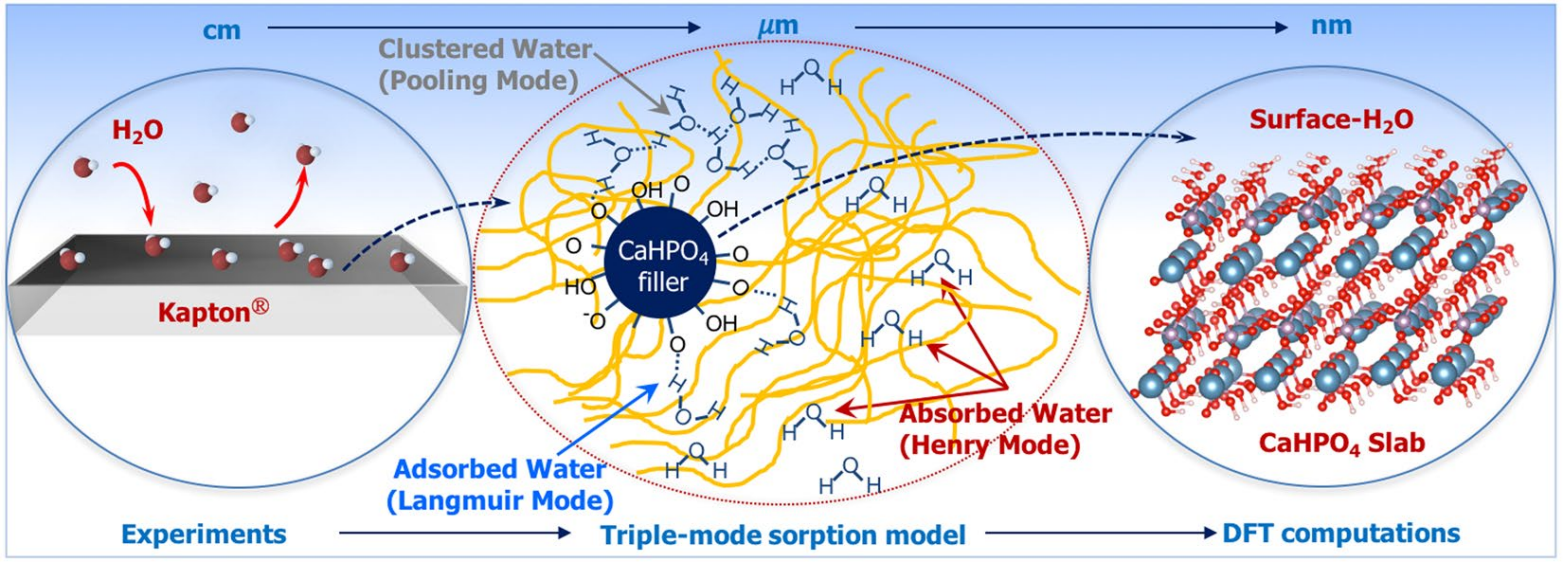
Schematics depicting the water-Kapton-filler interactions from macro-to-molecular levels (cm to nm scales) and the corresponding approaches used at each level.

In this study, we develop a new approach to explore moisture sorption phenomena by connecting macro, meso, and molecular scales through thermogravimetric experiments, mesoscale triple-mode sorption modeling, and first principles density functional theory (DFT) calculations. A schematic of this approach is shown in Fig. 1. Dynamic vapor sorption (DVS) experiments quantify the differences between the two materials and demonstrate a substantially larger absorption capacity (i.e., Henry’s mode sorption) and clustering or pooling for Kapton HN compared with Kapton H. Experimental results were analyzed using a triple-mode sorption model^16^ to establish mode-specific sorption contributions. Molecular-level DFT calculations were coupled with the first principles thermodynamics (FPT) to understand the interaction of moisture on CaHPO_4_ surfaces at realistic temperature and pressure conditions. Along with experiments and sorption-diffusion modeling, our molecular-level predictions for water binding affinity and phase diagrams for moisture adsorption on specific CaHPO_4_ crystal facets provide guidance for selective manufacturing of filled polymers and tuning moisture affinity.

## Results

### Continuum-scale moisture sorption experiments

**Figure 2:**
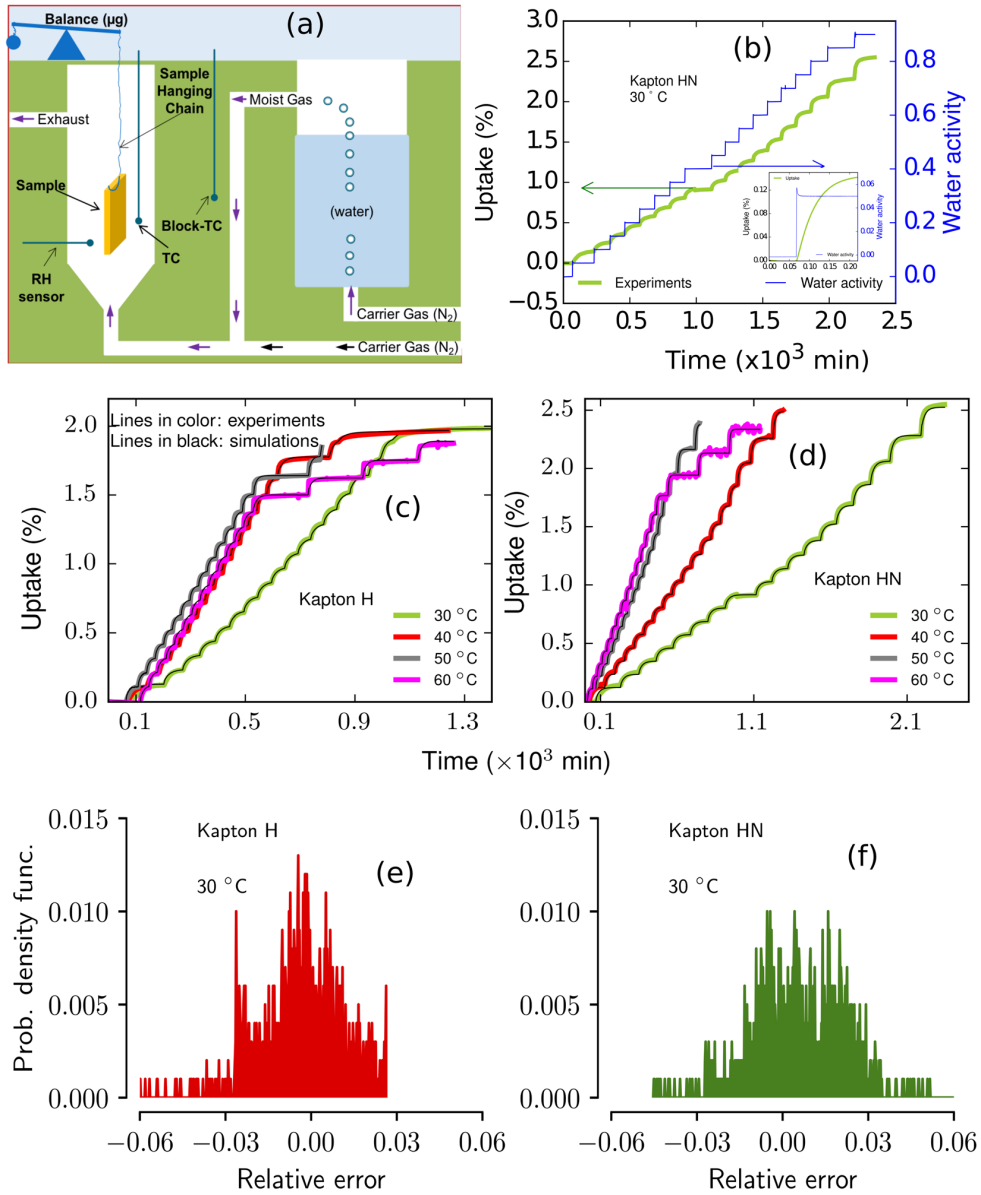
Panel a: Schematic showing the experimental setup with water reservoir, moist-gas flow system, and humidity control and gravimetric analysis; Panel b: moisture uptake (in wt. %) response (in left y-axis) to the programmed water activity steps (in right y-axis) in Kapton HN at 30 °C and a typical water activity experiment with 5% RH steps from 0 to 90% RH (in right y-axis); inset figure panel b: Magnification of the first step response from panel c showing the transient moisture uptake (in %, on left y-axis) vs. time (in minutes) or water activity (on right y-axis) profile in Kapton HN; Panels c and d: Experimental and model simulated moisture uptake (in %) vs. time for Kapton H and Kapton HN, respectively, at 30, 40, 50, and 60 °C; Panels e and f: Error probability distribution for experimental and model simulations at 30 °C Kapton H and Kapton HN, respectively, with 95% confidence interval. Experiments are shown using colored lines and model simulations are shown using black lines. Corresponding water activities of each experimental profile from panels e and f are not shown here. Water vapor activity from 0 to 0.9 corresponds to the relative humidity range of 0 to 90%.

Dynamic gravimetric type experiments were conducted over a wide range of water activities to quantify the moisture uptake in Kapton H and HN. A schematic of the experiment is provided in Fig. 2 (panel a) and a full description of the experiment is provided in the methods section. In a typical isotherm experiment, the sample is dried in the chamber, then exposed to a series of defined water activity steps (i.e., 5% RH per step) in a flow-gas environment under isothermal conditions (see Fig. 2, panel b, right y-axis). The sample mass change is recorded after each water activity step until the sample weight equilibrates, or the software reaches a pre-set time limit. Figure 2 panel b shows the water activity steps and moisture uptake profile for Kapton HN at 30 °C. Magnification of the first water activity step (see inset panel b, Fig. 2) clearly shows the transient moisture uptake profile after the relative humidity inside the chamber is increased. A small overshoot on the water activity at the beginning of each step is associated with the equipment PID control, however, no significant impact was observed on the moisture uptake profile. The duration at each water activity level is determined by the experimental software and coarse analysis of the uptake curve to determine if the material has equilibrated. As such, there are some steps in the isotherm that have longer time steps than others; these variations in duration do not alter the analysis and modeling, and do not necessarily imply any change in the sorption properties of the material.

We performed moisture isotherm experiments on Kapton and Kapton HN at four different temperatures between 0 and 90% RH, and the results are shown in Fig. 2 (panels c and d). Overall, moisture uptake profiles of both materials were very similar. Both Kapton H and HN exhibited maximum moisture capacities (at 90% RH) that were insensitive to temperature; this is consistent with the observation by Sacher *et al*.^35^. Both Kapton formulations uptake a significant amount of moisture relative to other polymeric materials such as unfilled polydimethyl siloxane (e.g., Sylgard-184^16^ takes up ~20 times less moisture) and high density polyethylene (e.g., ~200 times less moisture)^36^, and non-polymeric materials such as Zircar RS1200 (e.g., ~2 times less moisture)^16^. Finally, both formulations exhibit equilibrium isotherms (not shown) that are linear with respect to water activity over a wide range of humidities. This linear behavior suggests that the sorption behavior is primarily dominated by one sorption mechanism and will be discussed in the continuum modeling section.

The only observable difference between the moisture uptake data of the two Kapton formulations was the total moisture uptake. One can see that Kapton HN sorbs nearly 0.5 wt% more moisture than Kapton H at the maximum humidity. Several other differences are revealed in the continuum-scale modeling of these experiments.

### Continuum-scale sorption and diffusion modeling

Continuum scale modeling of the Kapton H and HN results using our triple-mode sorption model allows for further interpretation of sorption mechanisms and enables simulations of material response to other hygrothermal conditions. The triple-mode sorption model consists of three sorption models: Henry’s model, Langmuir model, and a pooling model. The model is described in the method section and previous publications^16,20^. In the model calibration, it is extremely important that the optimized parameter set globally minimizes the objective function used to describe the match between experiment and model.

**Figure 3:**
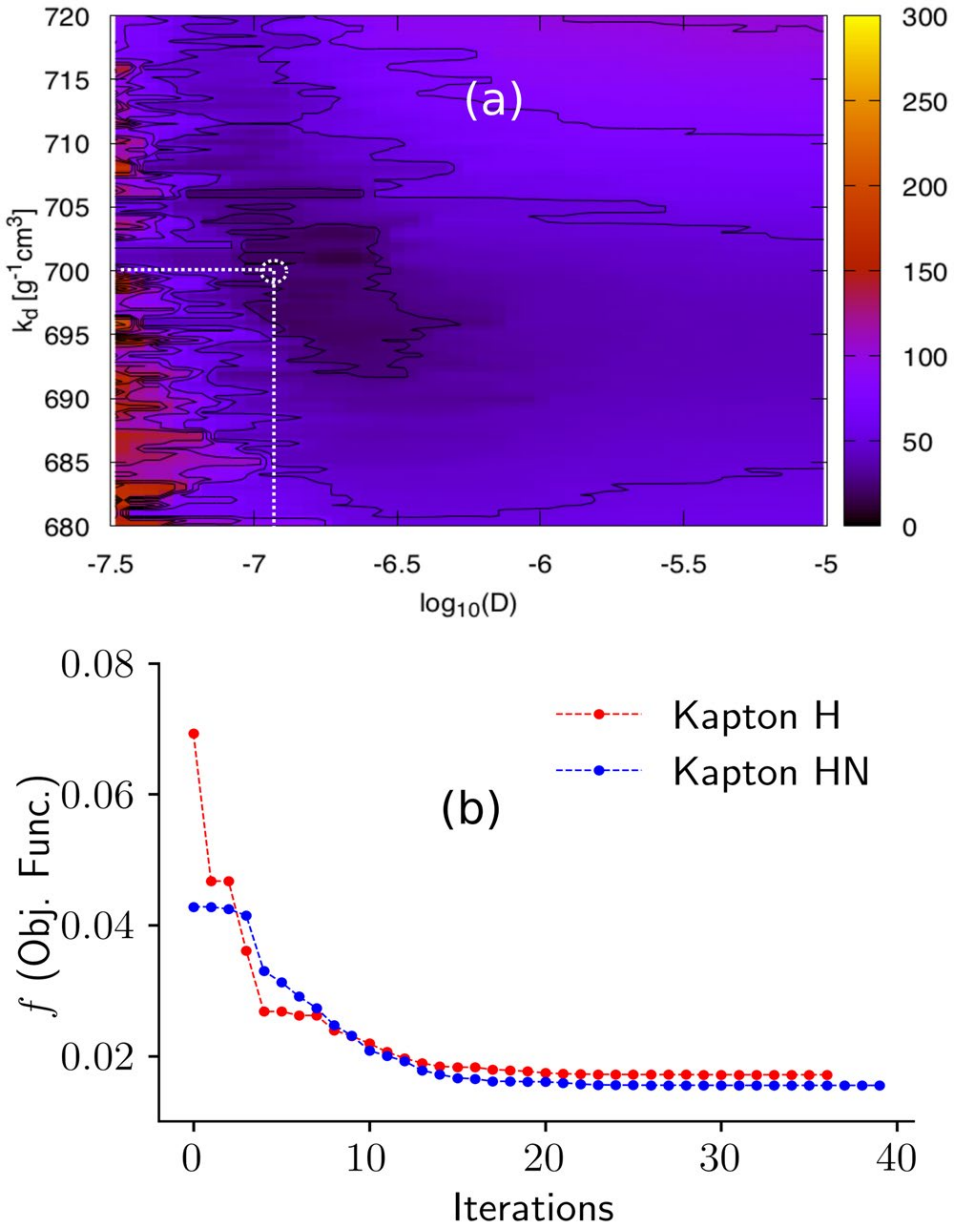
Panel a: Surface plot showing *k*_d_ and log(*D*) variation in terms of objective function and the global minimum (at the intersection of the dotted lines). Color bar for the objective function values is shown on the right. Panel b: Objective function evolution during SCE optimization of triple-mode sorption model for Kapton H and Kapton HN data at 30 °C. Optimization convergence criterion was set to *δE* = 1 × 10^−5^ for the relative change in the objective function.

The initial parameter calibration was performed using the PSUADE (Uncertainty Quantification code and sampling-based search)^4^. For both materials, the calibration was performed using the experimental data at all temperatures considered here. In this method, more than 1000 sample points were generated using Latin Hyper Cube sampling that were used to compute and compare the objective function. The PSUADE results for two parameters (*k*_d_ and *D*) are plotted in Fig. 3 (panel a) enabling visualization of the objective function landscape to identify the global minimum value and sensitivity for each parameter. Visualizing all the parameters on a single 2D plot is unfeasible, so sensitivity and parameter optimization were performed through non-visual analysis methods.

The PSUADE optimization only provides a coarse optimization. The calibrated parameters from PSUADE served as an initial guess for the Shuffled Complex Evolution (SCE) optimization method^37^ that was performed to obtain more precise and accurate model parameters for each sample. The PSUADE and SCE optimizations were performed using only the sorption experimental data (i.e., uptake only) at different temperatures. Figure 3 (panel b) shows the evolution of objective function during a SCE run for Kapton H and Kapton HN at 30 °C. Most of the parameters converged after 25–30 iterations as shown in panel a, Fig. 3. Moreover, the global minima between Henry’s constant (*k*_d_) and diffusivity (*D*) derived from the SCE optimization aligns with the PSUADE landscape, which confirms the robustness of the SCE optimization method. We note that these SCE optimizations are very computationally expensive even for the 1D simulations, and become cost prohibitive for high dimension (i.e., 2D or 3D) problems.

Simulations of the experimental conditions using the calibrated model are plotted with the experimental data in panels c and d, Fig. 2). One can see the excellent match between the calibrated model and the experimental results at all temperatures and water activities investigated (i.e., 30–60 °C and 0 to 0.9 water activity or dry to 90% RH). Figure 2 panels e and f show the relative error between model and experiment for the 30 °C results. In general, 95% of the error probability distribution falls within ±4% of error range and centered at zero (see further in Supplementary Information S3). This implies that the model performs well (i.e., no under or over prediction) against the experimental data. The ability to capture the entire experimental profile for moisture uptake (i.e., from low to high water activities) for both polymers validates our approach of using the triple-mode sorption model to simulate the moisture sorption-diffusion in these materials.

The SCE optimized parameters for 50 °C are listed in Table 1 and the parameter sets at all the other temperatures are provided in Supplementary Information (see Tables S1 and S2). Diffusivity values for Kapton samples across all temperatures were between ~1 × 10^−6^ and 2 × 10^−7^ cm^2^ min^−1^, which is consistent with previously reported values of ~1 × 10^−7^ ^14,35^. The diffusion activation energies, computed using an Arrhenius type relationship $D={D}_{o}\,\exp (\frac{\,-{E}_{a}}{RT})$, were in the range of 30–40 kJ/mol. Details are given in Supplementary Information S4. Our results show that the most sensitive parameter is Henry’s law constant (*k*_d_) in both Kapton H and HN.

**Table 1:** SCE optimized parameters for triple-mode sorption in Kapton H and Kapton HN at 50 °C.

Parameter	Symbol	Calibrated results at 50 °C	
		Kapton H	Kapton HN
Effective diffusivity	*D*	2.75 × 10^−7^	9.99 × 10^−7^
Desorption rate	*k*_s_	3.97 × 10^−1^	8.65 × 10^−1^
Langmuir capacity	${C^{\prime} }_{{\rm{H}}}$	5.0 × 10^−2^	1.0 × 10^−1^
Langmuir affinity	*b*′	2.676 × 10^−1^	2.342 × 10^−1^
Pooling factor	*α*	0.5 × 10^−1^	1.0 × 10^−1^
Pooling threshold	${C}_{\,{\rm{H}}}^{0}$	1.169 × 10^1^	1.193 × 10^1^
Pooling power	*n*	1.933	1.954
Henry’s law constant	*k*_d_	2.53 × 10^2^	2.70 × 10^2^

**Figure 4:**
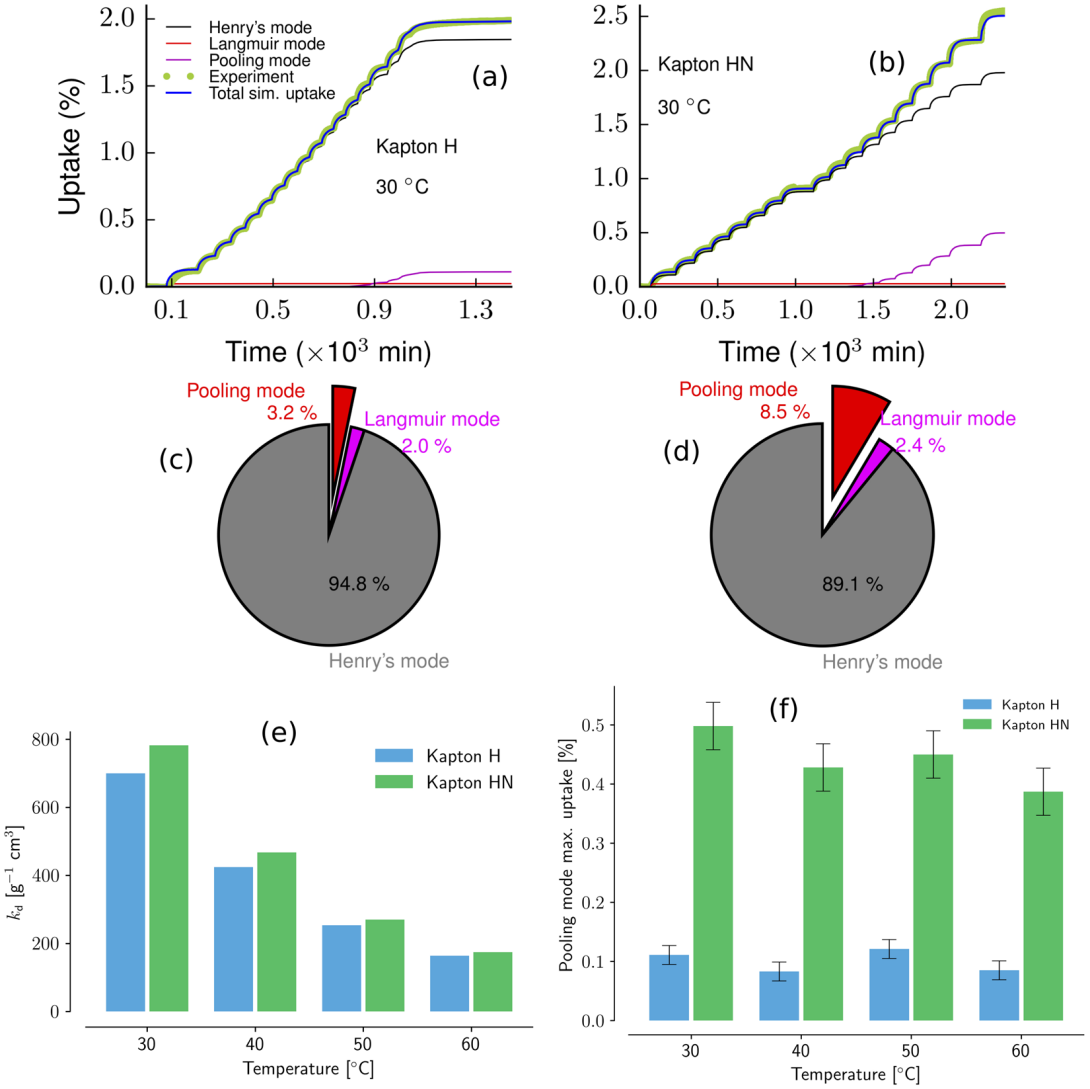
Triple-mode sorption analysis of Kapton H and Kapton HN. Panel a: moisture uptake (in %) vs. time for Kapton H at 30 °C; Panel b: Moisture uptake (in %) vs. time for Kapton HN at 30 °C; Panel c: Moisture uptake (in %) vs. time for Kapton H at 30 °C; Panel d: Moisture uptake (in %) vs. time for Kapton HN at 30 °C; Panel e: Comparison of Henry’s mode constant *k*_d_ of Kapton H and Kapton HN at temperatures of 30, 40, 50, and 60 °C; Panel f: Maximum percentage uptake at the water activity of 0.9 due to pooling mode in Kapton HN and Kapton H.

The optimized parameters enable further differentiation between Kapton H and HN sorption mechanisms. Figure 4 (panels a and b) show the constitutive sorption models (Henry, Langmuir and pooling), derived from the optimized model, in comparison to the full model and experimental uptake data. One can clearly see that both materials sorb a substantial amount of water via the Henry’s absorption mechanism and a negligible amount via Langmuir’s adsorption mechanism. The biggest difference between Kapton and Kapton HN is the amount of moisture that is sorbed via the pooling mechanism. More prominent distinctions can be seen when the percent contribution of each mode to the moisture uptake over the entire experiment is plotted as in panels c and d (Fig. 4). In Kapton HN, the pooling mode uptake is approximately 5 times higher than in Kapton H. Panels e and f (Fig. 4) show the variation of uptake due to the Henry’s law constant, *k*_d_ (at the highest water activity, i.e., 90% RH) and pooling at each temperature. It is evident that the pooling uptake is significantly higher in Kapton HN versus H at every temperature studied, which helps explain why the overall moisture uptake in Kapton HN is greater than H. However, Henry’s absorption also contributes substantially to the moisture uptake in these materials and Fig. 4 panel e demonstrates that the *k*_d_ of Kapton HN is always slightly greater than that of Kapton H. One can also see a decreasing trend with increase in temperature.

### Discussion of continuum-scale moisture uptake mechanisms

The sorption mechanisms for moisture uptake can be attributed to chemical and morphological properties of materials. In previous studies of filled and unfilled polydimethyl siloxane (PDMS), we observed significant moisture uptake via the Langmuir mechanism in the filled PMDS and very little Langmuir sorption in unfilled materials^16,20^. The PDMS matrix is typically hydrophobic and the filler in those studies (SiO_2_) was hydrophilic^38,39^. Hence, it is reasonable to conclude that the Langmuir sorption observed in filled-PDMS was due to moisture molecules van der Waals bonding to the hydrophilic sites on the filler.

The pooling of moisture in a material is associated with porosity. Pore filling of mesoporous materials involves large uptake of moisture due to the clustering mechanism and shows an exponential profile in an uptake versus water activity plot, especially at the higher water regions. For example, substantial pooling was observed in an alumina matrix composite material, Zircar RS1200, which has a high porosity (approximately 40%) and consists of alumina (82 wt% Al_2_ O_3_), silica (12 wt% SiO_2_), and other metal oxides (6%). The nucleation of pooling in Zircar is, most likely, founded on a monolayer of Langmuir sites, as this material also demonstrated significant Langmuir sorption^16^. Unfilled PDMS, which has very little porosity and negligible Langmuir sorption, is still able to cluster a small amount of moisture at elevated humidities^16,20^. However, once filler is added to the PDMS matrix, the pooling increases substantially^16,20^. It is likely that the silica filler in our filled-PDMS studies not only nucleated pooling via the Langmuir monolayer, but also created more porosity in the material thus providing the void volume necessary to enable pooling. Hence, fillers in materials may enable multiple different moisture sorption mechanisms by changing both the chemical and morphological nature of the material.

The key difference between Kapton H and HN is the presence of filler. Our analysis demonstrated that pooling (clustering) was present in both Kapton samples, but was much higher in Kapton HN. This is likely due to the CaHPO_4_ in Kapton HN. Langmuir sorption was relatively small in both Kapton H and Kapton HN, which was somewhat unexpected as we had hypothesized that CaHPO_4_ would add Langmuir sites to Kapton HN. An atomistic study was executed in order to further understand the nature of the moisture interactions with CaHPO_4_. Our atomistic modeling results, discussed below, indicate that the moisture interactions vary with the exposed crystal facets of CaHPO_4_. Unfortunately, we do not know the crystal morphology of CaHPO_4_ in Kapton HN and thus only tentatively conclude that the pooling uptake in Kapton HN is due to increased porosity created via the presence of filler.

### Atomistic modeling of CaHPO_4_ surfaces and H_2_O interactions

The continuum-scale modeling and experiments discussed above left many unanswered questions concerning the mechanisms and interactions of water molecules with the CaHPO_4_ filler in Kapton HN. Atomistic modeling provides qualitative and quantitative information about the CaHPO_4_ crystal structure and water-surface interactions. Our modeling relied on Density Functional Theory (DFT) calculations using the Vienna Ab-initio Simulation Package (VASP). A full description of the basis set, corrections, assumptions, and first principles thermodynamics method is provided in the Methods Section. All calculations correspond to equilibrium state results and enable a deeper understanding of the mechanisms and behavior the filler in Kapton HN.

**Figure 5:**
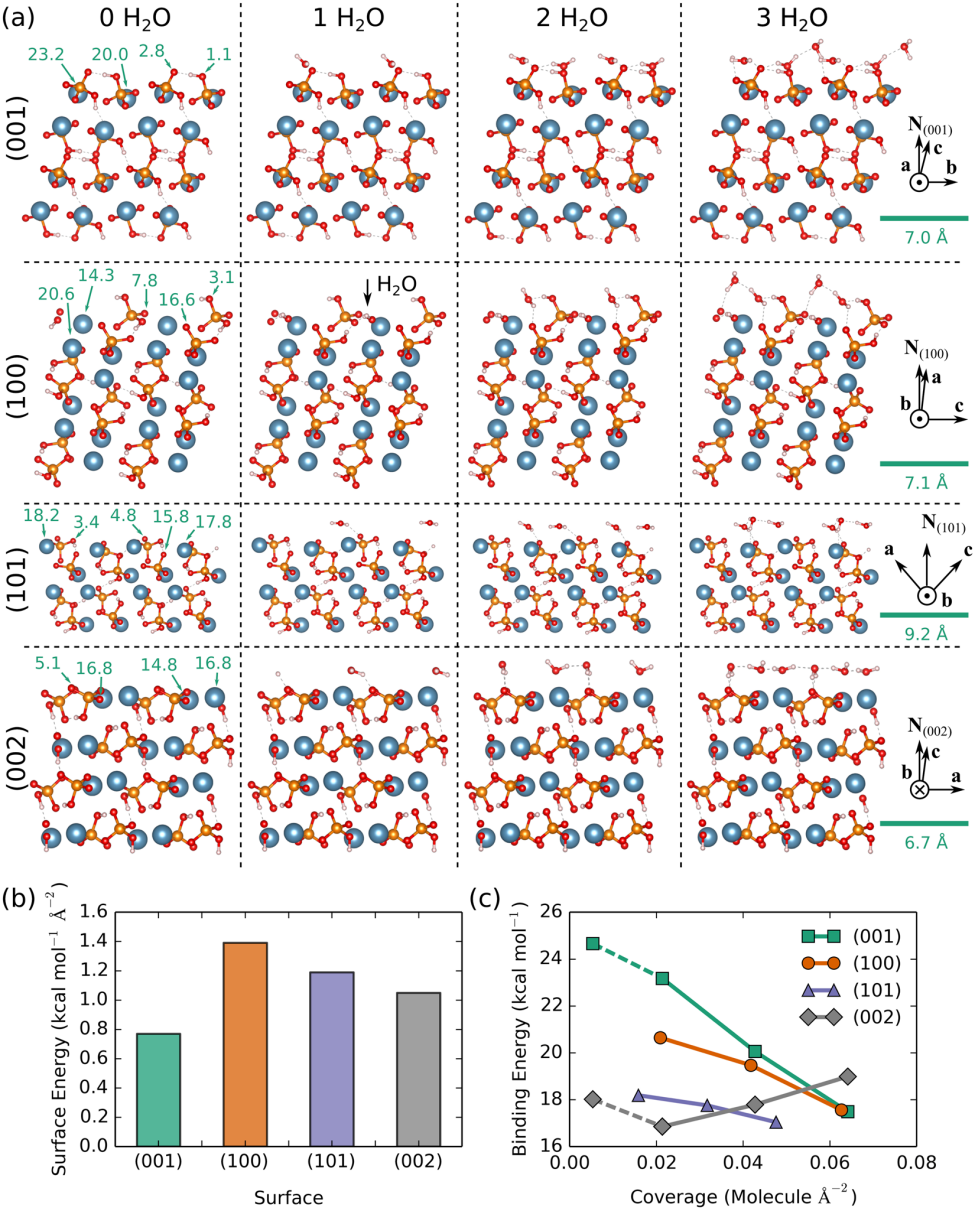
Panel a: Optimized configurations with exposed surface organized by row and number of adsorbed water molecules organized by column. Snapshots were rendered using VESTA^65^ with atoms colored blue, orange, red, and white for calcium, phosphorous, oxygen, and hydrogen. The top layer of atoms in each snapshot represents the material surface, lower layers are depth into the bulk material. Site specific binding energies (per H_2_O molecule) are displayed (in kcal/mol) on each clean surface shown in the first column and a Scale bar (in Å) is shown on the rightmost panel of each surface. Panel b: Surface energies computed using Eq. 14. Panel c: Average binding energy per adsorbed water molecule plotted as a function of coverage computed using Eq. 15.

CaHPO_4_ optimized surface configurations were obtained using DFT for slabs with exposed (001), (100), (101), and (002) facets and are shown in the first column of Fig. 5(a). These figures all show a cross-section or side view of the surface where the top layer is the surface and the three subsequent layers are below the surface and represent a bulk-material. The other columns show cases with adsorbed water molecules and are described in more detail below. A 2 × 2 repetition of the simulation cell in the transverse dimensions is shown in each snapshot and axes highlight relevant crystal directions and the normal vector **N**_(*ijk*)_ for the exposed face in each row. Surface energies computed using Equation 14 show that the stability of the four facets rank as (001) > (002) > (101) > (100) (see Fig. 5, panel b). Comparing snapshots of the optimized configurations reveals that the case with highest surface energy, namely (100), exhibits the “roughest” surface on a molecular level and one can see phosphate groups protruding up above the calcium layer intermittently. The two intermediate cases look quite similar, having a very smooth surface with calcium atoms directly exposed. The lowest energy case (001) has O and OH groups that protrude above the calcium sites creating a uniform surface. Note that because the surface energy for (001) is lower than that for (002), that the former would be preferred during crystal growth and one would not anticipate natural formation of (002) faces.

Energetically favorable sites for water adsorption were identified through a systematic, but non-exhaustive, search over each optimized surface configuration. Trial sites for water adsorption were identified on the crystal surfaces based on chemical intuition. For example, the polarizable water molecule was oriented to either (1) enable hydrogen bonding to O-groups on the surface or (2) enable the oxygen interactions with the calcium sites on the surface. At least two calcium sites and two oxygen sites were considered for each surface. Initial configurations were prepared from optimized surface configurations in which the water oxygen atom was placed directly over a surface calcium atom or in which one of the water O-H bonds was aligned in the *z* direction with the H directly above a surface phosphate oxygen atom. The lowest-energy optimized configuration with a single water adsorbed on each surface was used as the starting point for preparing initial configurations with two water molecules. A similar procedure was used to prepare surfaces with three water molecules. Snapshots of the lowest energy configurations are shown in Fig. 5(a).

The binding energy for water molecules adsorbed on each surface (*ijk*) was computed from Eq. 15 using the lowest energy configurations shown in columns two, three, and four in Fig. 5(a). The absolute value of the average binding energy per water molecule is plotted as a function of surface coverage in Fig. 5(c). In all cases, water binding is energetically favored, and larger absolute values indicate stronger binding. An extra set of calculations at very low coverage were performed for the two lowest surface energy cases, namely (001) and (002), using cells with a single water molecule adsorbed on a slab with a 2 × 2 transverse area. Linear interpolations between the data points for these two low-coverage cases and those points obtained using 1 × 1 cells are shown as dashed lines. A 2 × 2 × 1 k-space mesh was used for the 2 × 2 cells and a 4 × 4 × 1 k-space mesh was used for the 1 × 1 cells.

It is highly favorable for water molecules to adsorb on all four crystal facets. The predicted binding energy per adsorbed molecule ranges from 16.9 kcal mol^−1^ to 24.7 kcal mol^−1^, and is significantly stronger than the hydrogen bonds in liquid water^40^. The binding energy per molecule decreases with increasing coverage for the (001), (100), and (101) cases, which is expected. Surprisingly, the binding energy per molecule increases with increasing coverage in the (002) case. The (001) case has both the lowest surface energy and highest water binding energy over most of the coverage interval. To understand the electronic interactions involved with sorption, we examined the electronic density of states (DOS). Total DOS plots (shown in the Supplementary Information, Fig. S4) reveal a filling out of states near the highest occupied molecular orbital (HOMO) and a projected DOS analysis (Fig. S5) revealed peak shifting and broadening of the molecular orbitals localized to the surface Ca and water O atom involved in sorption. Significant overlaps of s- and p-like states centered on the Ca and O atoms, coupled with the particularly large binding energies, suggest a chemisorption process.

Comparison of the snapshots in Fig. 5(a) reveals that water-water hydrogen bonds form a concerted network on the (002) surface that likely increases the binding energy with increasing coverage. With two or more water molecules, one of the (002)-surface adsorbates forms two hydrogen bonds with phosphate oxygen atoms and also hydrogen bonds to adsorbate(s) on calcium sites. In the case with three water molecules, these hydrogen bonds form a nearly linear chain through the periodic boundary, which may be a consequence of the small simulation cell. However, as the case with two water molecules still exhibits increased stability without forming hydrogen bonds through the periodic boundary, it is reasonable to infer that water-water hydrogen bonds would contribute to the binding energy in larger (002) systems. In contrast, water molecules on the other surfaces do not arrange into highly ordered hydrogen bonding networks for the amounts of coverage considered here. It seems likely that the topology of calcium and oxygen sites on the (002) surface promotes interactions between multiple adsorbed water molecules.

The minimum in the binding energy in the (002) case can be rationalized in terms of simulation cell sizes and number of adsorbed water molecules. Both the 2 × 2 cell (coverage ≈ 0.005 molecules Å^−2^) and the 1 × 1 cell (coverage ≈ 0.021 molecules Å^−2^) have only one water molecule adsorbed, but the 2 × 2 surface has more degrees of freedom and can optimize through subtle surface reconstruction to a more tightly bound configuration. (A similar argument holds for the (001) case as well.) Meanwhile, increases in binding energy with increasing coverage are the result of interactions between adsorbate molecules. Cells for coverage > 0.03 molecules Å^−2^ contain more than one water molecule, and it is apparent that the (002) surface promotes strong water-water interactions leading to an increase in binding energy per molecule with increasing coverage. Because the search over adsorption sites was not exhaustive, it is not clear whether other calcium phosphate surfaces could also promote water-water interactions similar to (002).

### First principles thermodynamics and surface heterogeneity

**Figure 6:**
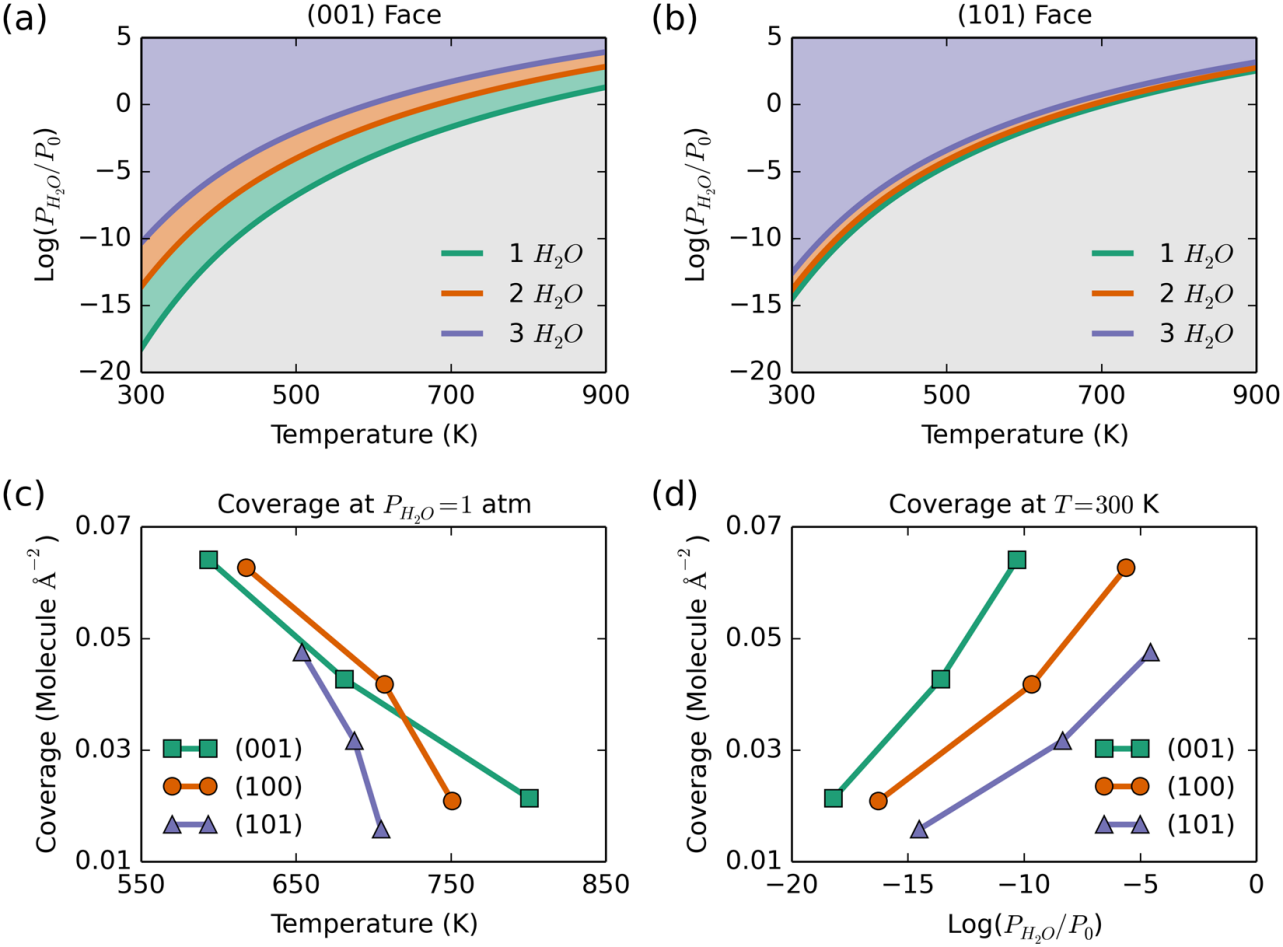
Panel a and b: First-principles predicted phase diagrams for water coverage on (**a**) (001) and (**b**) (101) surfaces of calcium phosphate crystals. Panel c: Temperature-dependent coverage at ${P}_{{{\rm{H}}}_{{\rm{2}}}{\rm{O}}}=1$ atm. Panel d: pressure-dependent coverage at *T* = 300 K for water adsorption on selected crystal facets.

Phase diagrams for water adsorption were obtained using first-principles thermodynamic calculations following the approach described in the Methods Section. Two representative cases are shown in Fig. 6 panels (a) and (b) for the (001) and (101) faces, respectively. The partial pressure of water ${P}_{{{\rm{H}}}_{{\rm{2}}}{\rm{O}}}$ is measured with respect to a reference pressure *P*_0_ = 1 atm. Four distinct regions can be seen in the phase diagram for (001) coverage. The first region, in gray, is free of water adsorbates and is favored at the lowest pressures and elevated temperatures. The green region represents the single water adsorption surface; one can determine the temperature and partial pressure conditions whereby the single water can be desorbed, and the material will be restored to a water-free surface. Increasing the partial pressure of water or decreasing the temperature favors surfaces with 2, then 3 surface waters per unit cell. The four phase regions are less distinct for the (101) case because the binding energy per molecule varies less with coverage than in the (001) case (see Fig. 6(c) and discussion below).

Isobaric and isothermal coverage trends were extracted from the phase diagrams for the (001), (100), and (101) surfaces and are shown in Fig. 6 panels (c) and (d). Under isobaric conditions at ${P}_{{{\rm{H}}}_{{\rm{2}}}{\rm{O}}}=1$ atm (Fig. 6(c)), one would have to heat the CaPHO_4_ to approximately 810 K in order to completely drive the water molecules off the surface. In contrast, one would need to heat the crystal to 710 K to drive all the water molecules off of the (101) surface. The isothermal response plot (Fig. 6(d)) shows that a (001) surface requires the lowest water partial pressure (and consequently higher vacuum condition) to remove all the water molecules. The (100) and (101) surfaces require less extreme vacuum conditions to completely dry the surface. Typical values for the vapor pressure of water at room temperature and pressure (${P}_{{{\rm{H}}}_{{\rm{2}}}{\rm{O}}}\approx 0.03$ atm) yield $\mathrm{log}({P}_{{{\rm{H}}}_{{\rm{2}}}{\rm{O}}}/{P}_{0})\approx -\,1.5$, which indicates that under those conditions one would expect significant amounts of water adsorbed on calcium phosphate crystal grains. Both the isobaric and isothermal phase diagrams reveal that it is significantly easier to remove water from (101) surfaces than from either (100) or (001) surfaces. Tailoring crystal growth conditions to favor expression of (101) surfaces would facilitate both temperature- and pressure-based desiccation processes.

## Summary and Conclusions

Moisture sorption and uptake capacity of materials directly influences their physical and chemical properties. A detailed understanding of the underlying mechanisms that are activated during moisture exposure can aid in the design of materials and systems and is needed to predict device lifetime and performance. This work explores two different polyimide polymers (i.e., Kapton H and Kapton HN). The key difference between the two materials is the presence of CaHPO_4_ in Kapton HN, which is added as a processing aid. Dynamic vapor sorption experiments were conducted at a range of temperatures (30–60 °C) in isothermal conditions by varying the water activity from 0 to 0.90 (i.e., relative humidities from dry to 90%). Kapton HN showed higher moisture uptake compared to Kapton H at all the temperatures considered here.

Triple-mode (Henry’s absorption, Langmuir adsorption, and pooling mechanisms) sorption and diffusion model was used to quantify the moisture uptake by these two materials. The model accurately captures the entire moisture uptake profile from dry to 0.9 water activity level at every temperature tested and the derived parameters are consistent with the diffusion parameters in the literature. Almost 90% of the total moisture uptake was due to Henry’s mode in both samples. Pooling of the water molecules was observed at elevated water activities (i.e., >0.65). Kapton HN showed significant increase in Pooling mode moisture uptake with 5 times higher weight percentage compared to Kapton H. This can be attributed to the CaHPO_4_ present in Kapton HN, which may increase porosity and is a potential site for water clustering. While Henry’s mode type sorbed water can be removed relatively easily due to weak interaction (physisorption) between water and material, water sorbed on materials due to Langmuir type interaction (chemisorption on filler) is more likely to create long term outgassing problems in many systems with moisture sensitive units as this is harder to remove from the surface. Thus, surface treatment (for example, hydrophobic functionalization of silica filler surface to remove active -OH surface group^41^) of filler materials can be implemented to reduce such interactions.

Atomistic modeling of H_2_O interactions with CaHPO_4_ surfaces revealed new molecular-level information and showed that the interaction strength was highly correlated to the surface facet anisotropy. DFT computations showed that the (001) face has the highest binding affinity to water. Unlike other typical systems (for example, H_2_O on fcc metal surfaces^42^), our results show that hydrogen bonding between H_2_O molecules on (002) surfaces leads to an increase in binding strength with increase in surface coverage. Finally, first principles thermodynamics calculations were used to construct phase diagrams for realistic temperature and pressure environments. These phase diagrams showed that the CaHPO_4_ surface will retain moisture at room temperature and atmospheric pressure conditions. We show that it is significantly easier to remove water from (101) surfaces than from other facets considered here. Thus, it may be possible to fine tune moisture sorption behavior through tailored crystal growth.

## Methods

### Experimental details

All experiments were conducted using the IGAsorp^43^ instrument, designed by Hiden Isochema, which is equipped with a high resolution micro-balance. The instrument has relative humidity regulation accuracy of 0.02%, weight resolution of 0.05 *μ*g, and temperature regulation accuracy of 0.01 °C. Details on the equipment and experimental setup can be found in prior publications^44,45^. Two different types of materials, i.e., Kapton H (typical sample dimensions: length = 8.89 cm, width = 2.54 cm, height = 0.00508 cm (2 MIL), and density = 1.38 g cm^−3^) and Kapton HN (typical sample dimensions: length = 5.08 cm, width = 2.159 cm, height = 0.00889 cm (3.5 MIL), and density = 1.42 g cm^−3^), were used for this comparative study. Sample dimensions were measured using high precision calipers from Swiss Precision Instruments, Inc.^46^. Sample density was computed utilizing the sample weight and measured sample volume. Prior to each experiment in the IGAsorp, each sample was preconditioned with dry nitrogen stream of 250 ml/min for several days until a stable mass was observed, which allowed us to obtain a true dry state sample and served as a baseline for the sample mass change. The water activity range of 0–0.9 (i.e., Relative humidity range of 0–90%) and temperature range of 30–70 °C were considered in this study. For a typical isotherm sorption study, the water activity was scanned with 0.05–0.1 step corresponding to 5–10% RH. The moisture uptake (in %) is defined as,

1 $$u=\frac{m-{m}_{0}}{{m}_{0}}\times 100$$

where *m*_0_, *m*, and *u* are the initial dry mass, instantaneous moist mass, and the percentage mass uptake, respectively.

### Diffusion-sorption model

The mass balance equation with diffusion, kinetic Langmuir adsorption, Henry’s absorption, and pooling sorption in a material can be written as^20^:

2 $$\frac{\partial C}{\partial t}=\frac{\partial ({C}_{{\rm{H}}}+{C}_{{\rm{L}}}+{C}_{{\rm{P}}})}{\partial t}=\nabla \cdot (D\nabla C)-\frac{d{C}_{{\rm{H}}}}{dt}-\frac{d{C}_{{\rm{P}}}}{dt}-{k}_{{\rm{a}}}\,SC+{k}_{{\rm{s}}}({C^{\prime} }_{{\rm{H}}}-S)$$

where *C* [mg g^−1^] is the mobile gas concentration in terms of sample bulk mass, *C*_H_ [mg g^−1^] is the mass concentration of the absorbed (i.e., Henry’s mode) gas component per unit of sample bulk mass, *C*_L_ [mg g^−1^] and *C*_P_ [mg g^−1^] are the concentrations in Langmuir and pooling (clustering) modes, respectively, *D* [cm^2^ min^−1^] is the effective diffusion coefficient, and *t* [min] is the time. The last two terms of Eq. (2) describe the kinetics of reversible Langmuir adsorption. *k*_a_ [min^−1^mg^−1^ g] and *k*_s_ [min^−1^] are absorption and desorption rates, *S* [mg g^−1^] is the concentration of empty Langmuir sites, and *C*′_H_ [mg g^−1^] is the Langmuir capacity constant. The Henry’s mode concentration, *C*_H_, is treated as a mobile species due to its linear dependence on gas phase (mobile) concentration.

3 $${C}_{{\rm{H}}}={k^{\prime} }_{{\rm{d}}}\,C=\frac{{k^{\prime} }_{{\rm{d}}}\varphi }{{\rho }_{{\rm{b}}}}c={k}_{{\rm{d}}}\,c,$$

in which ${k}_{{\rm{d}}}={k^{\prime} }_{{\rm{d}}}\varphi /{\rho }_{{\rm{b}}}$ (cm^3^ g^−1^) is the Henry’s law constant used in simulations, *k′*_d_ is the dimensionless Henry’s law constant, *ϕ* is the porosity, *ρ*_b_ [g cm^−3^] is the bulk density, *c* [mg cm^−3^] is the gas-phase concentration of vapor. With the treatment of Henry’s mode as a mobile species, Eq. (2) can be expressed in terms of *C*_H_

4 $$\frac{1}{{k}_{{\rm{d}}}{\rho }_{{\rm{b}}}}\frac{\partial {C}_{{\rm{H}}}}{\partial t}=\nabla \cdot (D\frac{1}{{k}_{{\rm{d}}}{\rho }_{{\rm{b}}}}\nabla {C}_{{\rm{H}}})-\frac{d{C}_{{\rm{H}}}}{dt}-\frac{d{C}_{{\rm{P}}}}{dt}-\frac{{k}_{{\rm{a}}}}{{k}_{{\rm{d}}}{\rho }_{{\rm{b}}}}\,S{C}_{{\rm{H}}}+{k}_{{\rm{s}}}\,({C^{\prime} }_{{\rm{H}}}-S\mathrm{).}$$

When ${k}_{{\rm{d}}}$ ${\rho }_{{\rm{b}}}$ $\gg$ 1, Eq. 4 can be written as

5 $$\frac{\partial {C}_{{\rm{H}}}}{\partial t}=\nabla \cdot (D\,e\nabla {C}_{{\rm{H}}})-\frac{d{C}_{{\rm{P}}}}{dt}-{k^{\prime} }_{{\rm{a}}}SC+{k}_{{\rm{s}}}({C^{\prime} }_{{\rm{H}}}-S)$$

where *D*_e_ = *D*/*k*_d_*ρ*_b_ and *k*_a_ = *k*_a_/*k*_d_*ρ*_b_.

In the Langmuir sorption mode, the Langmuir affinity constant b′ (in mg^−1^ g) can be defined as,

6 $$b^{\prime} =\frac{{k}_{{\rm{a}}}}{{k}_{{\rm{s}}}}\mathrm{.}$$

The equilibrium pooling concentration, *C*_P_ (in mg g^−1^), is a nonlinear function of Henry’s mode local concentration and is defined as:

7 $${C}_{{\rm{P}}}=\frac{{K^{\prime} }_{{\rm{C}}}{({k}_{{\rm{d}}}{C}_{{\rm{H}}})}^{n}}{n}=\alpha {C}_{{\rm{H}}}^{{\rm{n}}}$$

where ${K^{\prime} }_{{\rm{C}}}$ is the equilibrium constant for a clustering reaction, *k*_d_ is the Henry’s law constant, *α* is the lumped pooling coefficient, and *n* represents the number of molecules in each pool and is treated as a fitting parameter at continuum-scale. The lumped pooling coefficient *α* is defined as,

8 $$\alpha =\frac{{K^{\prime} }_{{\rm{C}}}{k}_{{\rm{d}}}^{{\rm{n}}}}{n}\mathrm{.}$$

The pooling concentration is further expressed as:

9 $${C}_{{\rm{P}}}=\alpha  {\mathcal H} ({C}_{{\rm{H}}},\,{C}_{{\rm{H}}}^{o})\,{({C}_{{\rm{H}}}-{C}_{{\rm{H}}}^{o})}^{{\rm{n}}}$$

where ${C}_{{\rm{H}}}^{o}$ (in mg g^−1^) is the threshold value of Henry’s concentration, at which the Pooling mode starts. ${\mathcal H}$ is the heaviside step function, which is expressed as^20^:

10 $${\mathscr{H}}=\{\begin{array}{cc}1, & {C}_{{\rm{H}}} > {C}_{{\rm{H}}}^{o}\\ 0, & {\rm{o}}{\rm{t}}{\rm{h}}{\rm{e}}{\rm{r}}{\rm{w}}{\rm{i}}{\rm{s}}{\rm{e}}\end{array}$$

To account for a decreased effective diffusion once pooling begins, a reduced tortuosity^20^ parameter *τ* was introduced at the point at which pooling started; below this point the tortuosity was 1.0. The effective diffusivity can be calculated as,

11 $$D={D}_{o}\tau $$

where *D* is effective diffusion coefficient, *D*_*o*_ is molecular-weight-dependent diffusivity, and *τ* is medium-specific tortuosity, which is a measure of the connectivity of pores and defined as the chord-arc ratio (ratio of the straight distance to the integrated length of the tortuous pathway).

### Parameters estimation and optimization

Model parameters are estimated using the uncertainty quantification code PSUADE^4,47^ and calibrated using the shuffled complex evolution (SCE) method^48^. First, a sampling based non-intrusive Latin Hypercube (LH) sampling method^49^ is used to generate a large number of sample points; sufficiently large to represent the parametric space. Each ‘sample point’ consists of a vector of all parameters (i.e., *D*, *k*_s_, ${C^{\prime} }_{{\rm{H}}}$, *b*′, *α*, *n*, and *k*_d_) in our model. To be consistent with the equilibrium Langmuir formulation, we use the Langmuir affinity constant $b^{\prime} ={k}_{{\rm{a}}}/{k}_{{\rm{s}}}$ [mg^−1^ g] instead of *k*_d_ in our parameter calibration. Then, each sample point is used to parametrize the model and the corresponding objective function is computed. Sample points resulting the smallest minimization function are chosen to be the candidates for the parameter optimization using the SCE^48^ method implemented in MATLAB^50^ with lower and upper bounds of all parameters defined. The objective function used for model calibration is:

12 $$f={\int }_{t}|m(t)-\hat{m}(t)|dt$$

in which *m* and $\hat{m}$ are the experimental and simulated mass uptake, respectively. The model simulated mass uptake is calculated as:

13 $$\hat{m}={\rho }_{b}\,{A}_{0}{\int }_{0}^{L}\,({C}_{{\rm{H}}}+{C}_{{\rm{L}}}+{C}_{{\rm{P}}})\,dx,$$

where *ρ*_b_ and *A*_0_ are the bulk sample density and sample area, respectively. SCE in PSUADE is used to find the best fit of model to experimental data with a set of best fit parameters. Our SCE optimization convergence criterion was set to 1 × 10^−5^ for the relative change in the objective function. Once the convergence criterion has been met, the optimization is set to complete and the final parameters are obtained. Our model parameters are set to be accurate within the error margin of 0.01%.

### Atomistic calculations

#### Density functional theory

Density functional theory^51,52^ (DFT) calculations were performed to characterize water adsorption on selected crystal facets of calcium phosphate using the Vienna Ab-initio Simulation Package^53,54^ (VASP) and three-dimensionally periodic simulation cells. All DFT calculations were performed using the Perdew-Burke-Ernzerhof^55^ (PBE) generalized gradient approximation functional with projector-augmented wave (PAW) pseudo-potentials^56,57^ and a 500 eV plane wave cutoff. The electronic structure was evaluated with spin polarization and Fermi-Dirac thermal smearing^58^ with the electron temperature set to 0.1 eV. Monkhorst-Pack^59^ k-space meshes were used and grids for specific simulation cells are given in text. Including Grimme D3 dispersion corrections^60^ yielded similar water adsorption binding energies to calculations without dispersion corrections, so only results without dispersion corrections are reported. The self-consistent field accuracy threshold was set to 10^−5^ eV and optimizations of the ionic degrees of freedom were performed with a force-based accuracy threshold of 10^−2^ eV Å^−1^.

#### Bulk and surface structures

We used the archived Materials Project calcium phosphate unit cell, which is triclinic (P1 space group) and contains four calcium atoms and four phosphate (HPO_4_) molecules (28 atoms total)^61^. Lattice parameters at *T* = 0 K and *P* = 0 atm were determined by optimizing a simulation cell containing the unit cell using a 4 × 4 × 4 k-space mesh. The optimized parameters are *a* = 6.715 Å, *b* = 6.971 Å, $c=7.086$ Å, $\alpha ={75.72}^{{\rm{o}}}$, $\beta ={83.31}^{{\rm{o}}}$, and $\gamma ={88.21}^{{\rm{o}}}$, with the maximum deviation from the archived structure being −2% for lattice parameter *c*. This optimized unit cell was used as a starting point for all subsequent simulation cell constructions. Simulation cells containing crystal slabs with exposed surfaces were constructed using the generalized crystal-cutting method^62^. Four crystal facets were considered, namely (001), (002), (100), and (101). Note that because the crystal is not centrosymmetric, the facets (*ijk*) and $(\bar{i}\bar{j}\bar{k})$ are not equivalent. While a given slab has both (*ijk*) and $(\bar{i}\bar{j}\bar{k})$ facets exposed, only the four facets listed above are considered in the water adsorption calculations. The crystal slabs were oriented so that the normal vector for the exposed crystal face was aligned in the *z* direction and a 15 Å vacuum region was added along that same direction. The (001), (002), and (100) slabs were two unit cells thick along *z* and a single unit cell wide in the two transverse dimensions, yielding 56 atoms in total. The smallest possible cell with an exposed (101) surface is twice as large as the primitive unit cell, so the corresponding (101) slab also contained 56 atoms. Atoms in the bottom half of the slab were held in fixed positions during all optimizations. Fixed-atom assignments were made on a molecular basis so that the HPO_4_ molecular configurations were either completely rigid or flexible. Calculations involving the slab simulation cells were performed with a 4 × 4 × 1 k-space mesh and with dipole corrections^63^ applied in the *z* direction.

#### First principles thermodynamics

Surface energies were computed from DFT energies for the optimized slab configurations and bulk crystal as

14 $${\gamma }_{{\rm{Surface}}}^{(ijk)}=\frac{({E}_{{\rm{Slab}}}^{(ijk)}-N{E}_{{\rm{Bulk}}})}{2A},$$

where ${E}_{{\rm{Slab}}}^{(ijk)}$ is the energy of the slab with exposed (*ijk*) and $(\bar{i}\bar{j}\bar{k})$ faces, *N* is the number of unit cells in the slab, *E*_Bulk_ is the energy of the bulk crystal unit cell without exposed surfaces, and *A* is the transverse area of the slab simulation cell. The total binding energy for ${N}_{{{\rm{H}}}_{{\rm{2}}}{\rm{O}}}$ water molecules adsorbed on a particular calcium phosphate surface was computed as

15 $${E}_{{\rm{Bind}}}^{(ijk)}={E}_{{\rm{Slab}}+{{\rm{H}}}_{{\rm{2}}}{\rm{O}}}^{(ijk)}-{E}_{{\rm{Slab}}}^{(ijk)}-{N}_{{{\rm{H}}}_{{\rm{2}}}{\rm{O}}}{E}_{{{\rm{H}}}_{{\rm{2}}}{\rm{O}}},$$

where ${E}_{{\rm{Slab}}+{{\rm{H}}}_{{\rm{2}}}{\rm{O}}}^{(ijk)}$ is the energy for the slab with adsorbed water molecules and ${E}_{{{\rm{H}}}_{{\rm{2}}}{\rm{O}}}$ is the energy for an isolated water molecule. Here, the energy ${E}_{{\rm{Slab}}}^{(ijk)}$ for the slab without adsorbates is taken as the reference. The energy ${E}_{{{\rm{H}}}_{{\rm{2}}}{\rm{O}}}$ was obtained for an optimized water molecule in a large 10^3^ Å^3^ cell computed with dipole corrections in all three Cartesian directions. First-principles thermodynamics calculations were performed to extract the phase diagram for water sorption as a function of temperature and pressure. The Gibbs free energy can be obtained from the binding energy, a vibrational contribution ${\rm{\Delta }}{F}_{{\rm{Vib}}}$, and the change in chemical potential for water ${\rm{\Delta }}{\mu }_{{{\rm{H}}}_{{\rm{2}}}{\rm{O}}}(T,\,{P}_{{{\rm{H}}}_{{\rm{2}}}{\rm{O}}})$ as

16 $${\rm{\Delta }}{G}^{(ijk)}(T,\,{P}_{{{\rm{H}}}_{{\rm{2}}}{\rm{O}}})=\frac{1}{2A}[{E}_{{\rm{Bind}}}^{(ijk)}+{\rm{\Delta }}{F}_{{\rm{Vib}}}-{N}_{{{\rm{H}}}_{{\rm{2}}}{\rm{O}}}\,{\rm{\Delta }}{\mu }_{{{\rm{H}}}_{{\rm{2}}}{\rm{O}}}(T,\,{P}_{{{\rm{H}}}_{{\rm{2}}}{\rm{O}}})]\mathrm{.}$$

The free energy depends on the temperature *T* and the partial pressure for water ${P}_{{{\rm{H}}}_{{\rm{2}}}{\rm{O}}}$. Vibrational contributions evaluated within the quasi-harmonic approximation were found to be very small (1 kcal mol^−1^) even near the maximum at *T* = 0 K, so they were omitted in the calculation of ${\rm{\Delta }}{G}^{(ijk)}(T,\,{P}_{{{\rm{H}}}_{{\rm{2}}}{\rm{O}}})$. The ${\rm{\Delta }}{\mu }_{{{\rm{H}}}_{{\rm{2}}}{\rm{O}}}(T,\,{P}_{{{\rm{H}}}_{{\rm{2}}}{\rm{O}}})$ represent the temperature and pressure dependent chemical potential term. Chemical potential of H_2_O can be calculated as:

17 $${\mu }_{{{\rm{H}}}_{2}{\rm{O}}}(T,\,{P}_{{{\rm{H}}}_{2}{\rm{O}}})={E}_{{{\rm{H}}}_{2}{{\rm{O}}}^{DFT}}+{\rm{\Delta }}{\mu }_{{{\rm{H}}}_{2}{\rm{O}}}(T,\,{P}_{{{\rm{H}}}_{2}{\rm{O}}}),$$

which contains the DFT computed energy of an isolated H_2_O molecule and chemical potential with temperature and pressure dependence (computation details can be found in refs^42,64^).

## Electronic supplementary material

Supplementary Information
