# Supplementary material for: Role of filler and its heterostructure on moisture sorption mechanisms in polyimide films
Source: Sci Rep. 2018 Nov 15;8:16889. doi: 10.1038/s41598-018-35181-1 (PMC6237878; doi:10.1038/s41598-018-35181-1)
Supplement: Supplementary file 1 — Supplementary Information [file 41598_2018_35181_MOESM1_ESM.pdf]

Supplementary information for

Role of filler and its heterostructure on moisture sorption mechanisms in polyimide films

Hom N. Sharma[1], Matthew P. Kroonblawd[1], Yunwei Sun[1], and Elizabeth A. Glascoe[1,*]
[1]Lawrence Livermore National Laboratory, 7000 East Ave., Livermore, California 94550, United States

[*]glascoe2@llnl.gov

S1. SCE optimized parameters for Kapton H at 30, 40, 50, and 60 °C.

SN	Parameters	Symbol	Calibrated results			
			30 ℃	40 ℃	50 ℃	60 ℃
1	Effective diffusivity	D	1.301×10^{-7}	1.43×10^{-7}	2.75×10^{-7}	2.10×10^{-7}
2	Desorption rate	k_s	2.06×10^{-1}	5.97×10^{-1}	3.97×10^{-1}	1.046
3	Langmuir capacity	C_H'	2.37×10^{-1}	5.0×10^{-2}	5.0×10^{-2}	5.0×10^{-2}
4	Langmuir affinity	b'	15.01	25.008	26.76	21.87
5	Pooling factor	α	5.029	5.00	5.00	5.00
6	Pooling threshold	C_H^0	12.252	12.257	11.69	12.57
7	Pooling power	n	2.217	2.857	1.933	2.10
8	Herny's law constant	k_d	699.997	424.365	253.62	163.603

S2. SCE optimized parameters for Kapton HN at 30, 40, 50, and 60 ℃.

SN	Parameters	Symbol	Calibrated results			
			**30 ℃**	**40 ℃**	**50 ℃**	**60 ℃**
1	Effective diffusivity	D	3.68×10^{-7}	6.12×10^{-7}	9.99×10^{-7}	1.33×10^{-7}
2	Desorption rate	k_s	8.6×10^{-1}	2.8×10^{-1}	8.65×10^{-1}	1.66×10^{-1}
3	Langmuir capacity	C_H'	1.0×10^{-1}	1.0×10^{-2}	1.0×10^{-2}	5.0×10^{-2}
4	Langmuir affinity	b'	28.21	25.50	23.42	27.07
5	Pooling factor	α	10.0	10.0	10.0	5.00
6	Pooling threshold	C_H^0	12.34	12.67	11.93	10.0
7	Pooling power	n	2.24	2.186	1.954	2.32
8	Herny's law constant	k_d	782.70	467.87	270.09	174.56

S3. Probability density plot for error analysis in Kapton H (panel a) and Kapton HN (panel b). Relative error (computed using relative error = (model result-experimental data)/(experimental data)) is relatively small and centered between ±4% indicative of an excellent match between experiment and simulations as shown in figure below. Analysis corresponds to Fig. 2 in the main text.

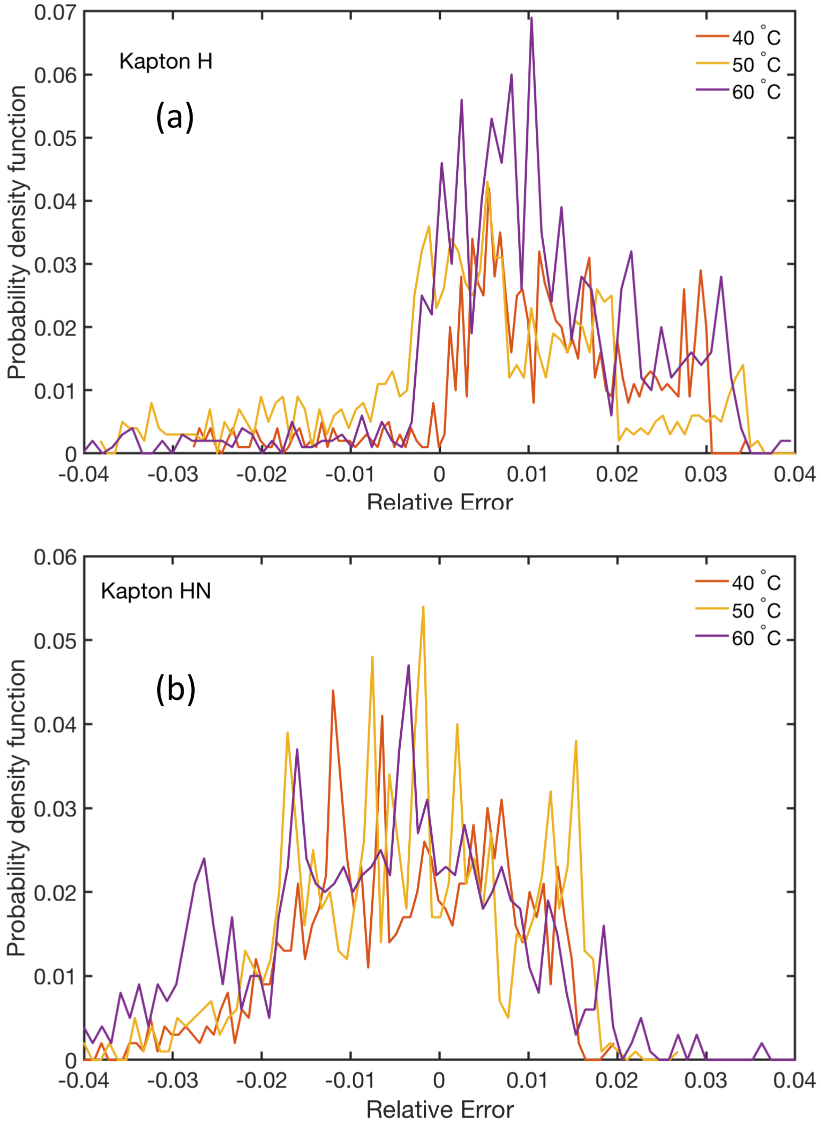

Fig S1: Probability density plot for error analysis.

S4. Diffusion coefficient comparison and activation energy calculation

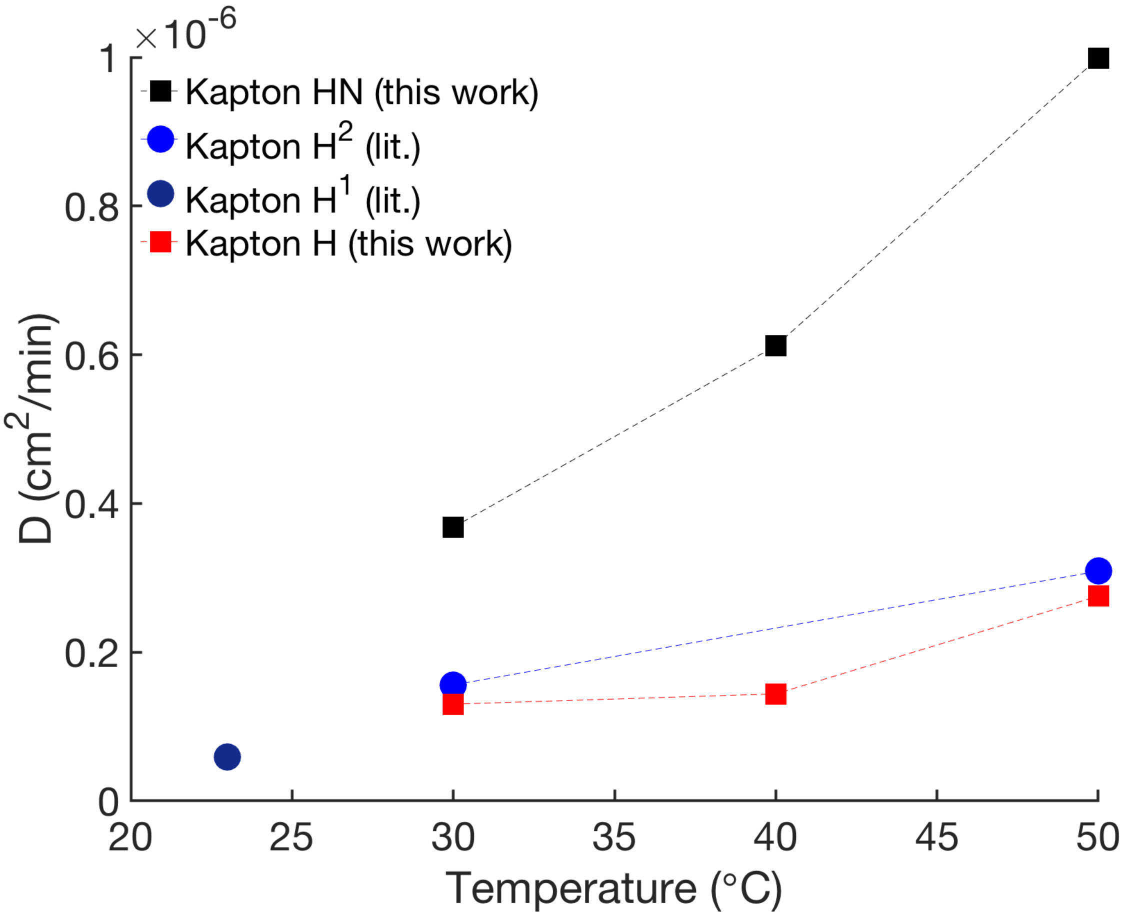

Fig S2: Comparison of diffusion coefficients for Kapton H and Kapton HN. Kapton H with subscript 1 and 2 data is taken from literature. [1, 2] Our optimized parameters are set to be accurate within the error margin of 0.01%.

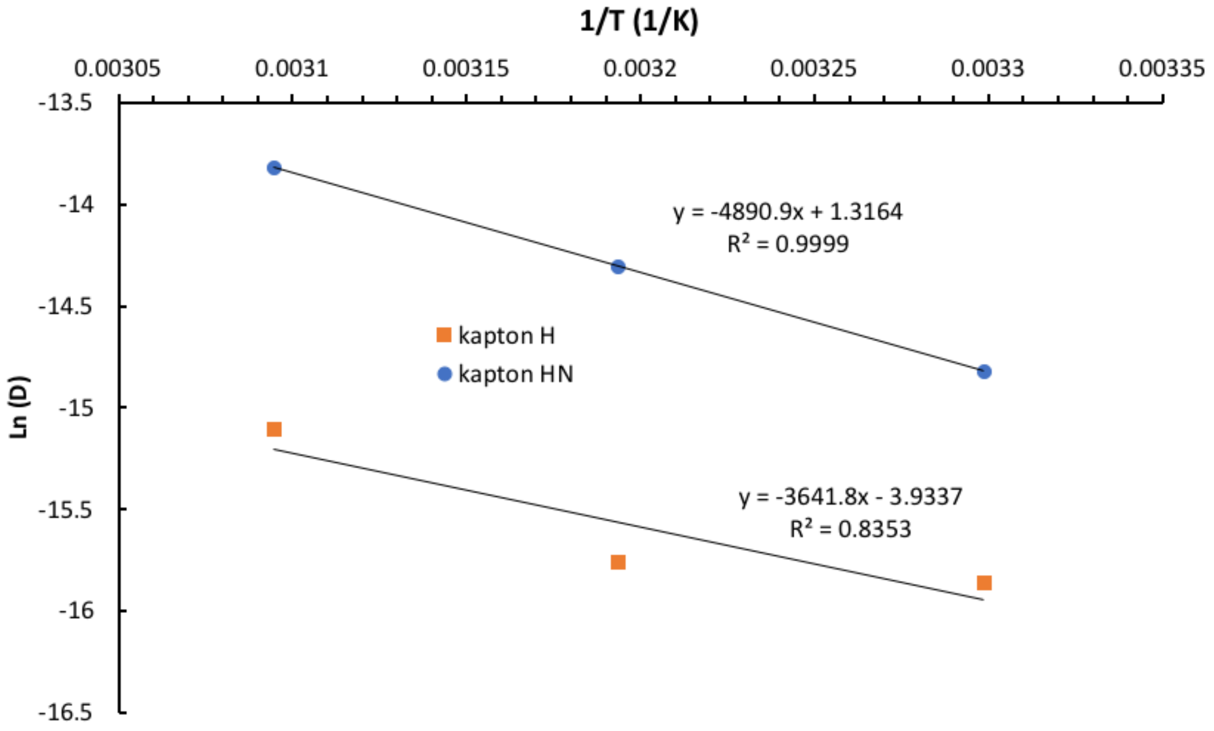

Fig. S3: Arrhenius plot of D, the solid line is a fit to an Arrhenius model $D = D_o \exp\left(\frac{E_a}{RT}\right)$. Activation energies for moisture diffusion are computed from the slopes (Kapton H Diffusion E_a = 30.3 kJ/mol and Kapton HN Diffusion E_a = 40.6 kJ/mol).

S5. Electronic density of state (DOS).

We computed the total Density of states (tDOS) and partial density of states of chosen surfaces. For pDOS, we decomposed the electron density and wave function into contributions from atomic orbitals, i.e., s, p and d orbitals centered on each atom. These DOS correspond to the gamma point and the single point calculation was performed at the gamma point only. The HOMO energy level has been set to zero for each curve. Figure S3 (total DOS) shows the filling out of states by shifting to right near the HOMO energy.

Figure S4 shows the projected atomic DOS of atom/molecule in an isolated state along with the pure slab surface in the lower part and the adsorbed states of those in the upper part. Results show the oxygen 2s and 2p orbitals attached to metal showed more shifting and broadening. Similarly, Calcium 4s and 3d orbitals showed peak shifting and broadening. Mostly 4s and 3p orbitals of Ca interacted with oxygen 2s and 2p orbitals (0 to -5 eV and -18- to -20 eV region). Such interaction is evident that the surface interaction between $\mathrm{CaHPO_4}$ and $\mathrm{H_2O}$ is chemisorption type.

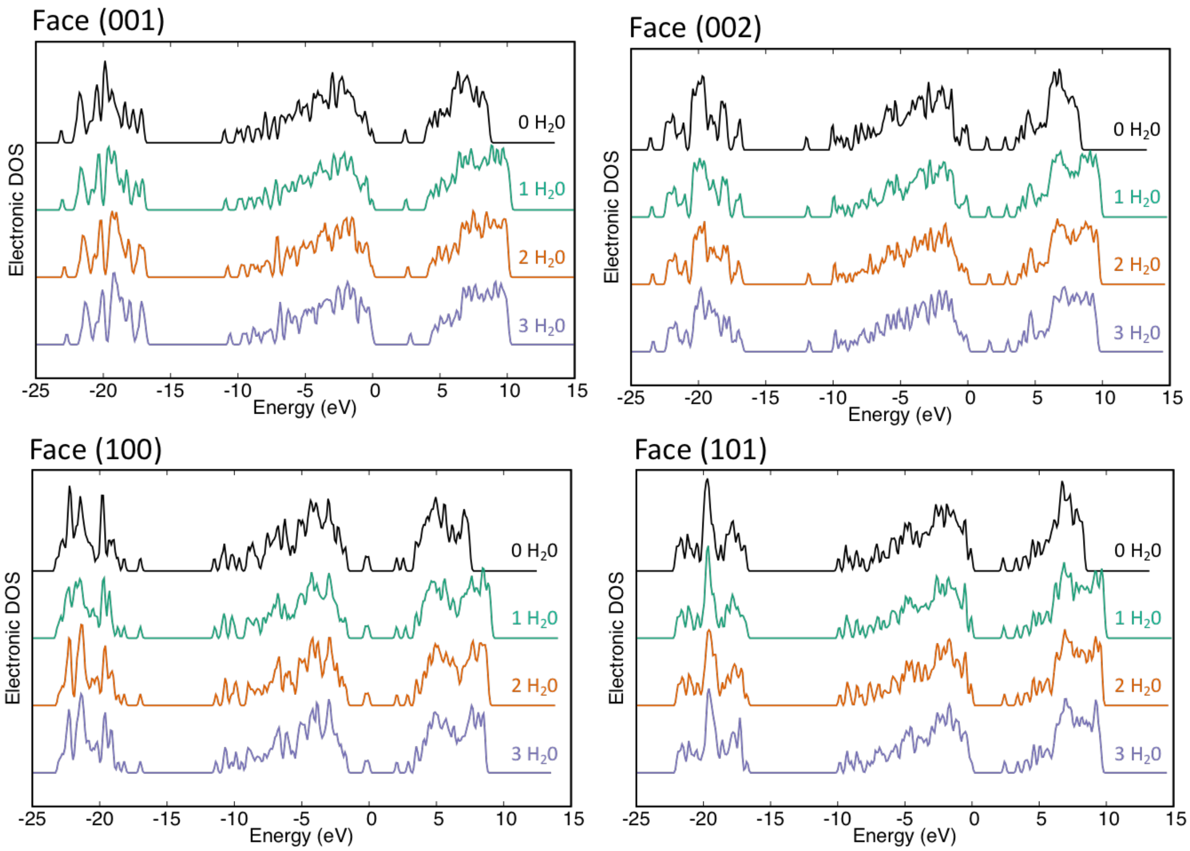

Fig. S4: total density of states computed using pristine (001), (002), (100), and (101) facets of $CaHPO_4$ and the subsequent adsorption of H_2O molecules on the same surfaces. E_{HOMO} energy was set to zero in all cases.

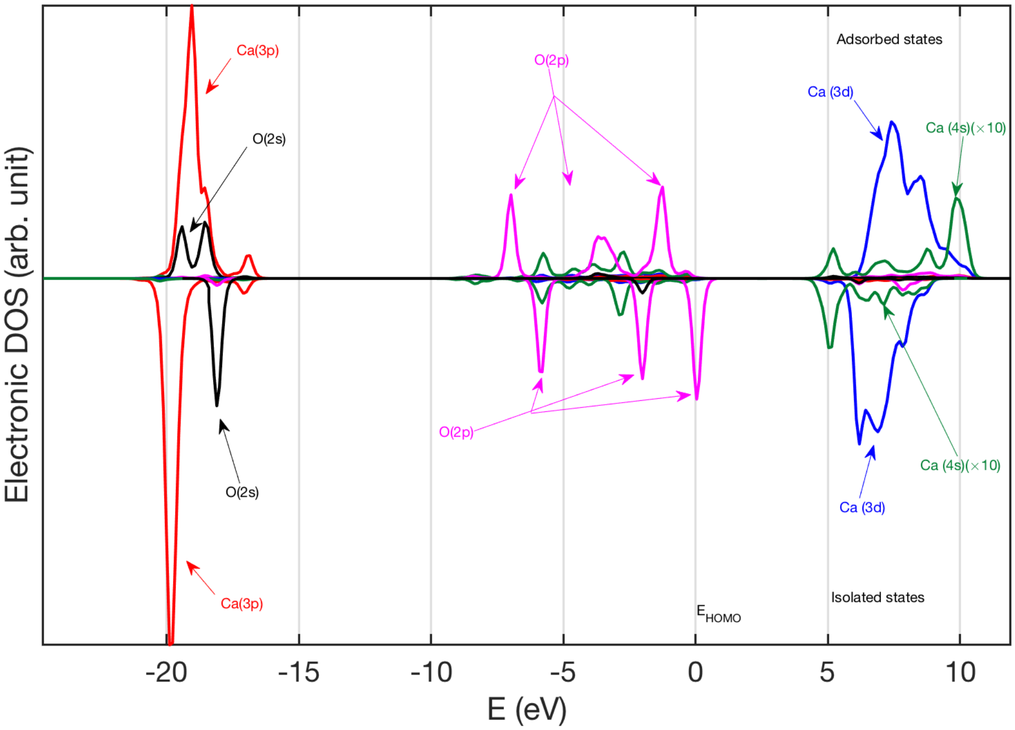

Fig S5: Projected density of states (P-DOS) analysis of adsorbed H_2O molecule on $CaHPO_4$ surface. The lower panel represents the projected DOS of Ca and O atomic orbitals in isolated atomic/molecular/pure slab state and the upper part represents the same in adsorbed state. Ca (4s) orbitals intensity was scaled by a factor of 10 to make them visible in the plot. HOMO energy (E_{HOMO}) is adjusted to zero in the plot.

Reference:

1. Han, H., Gryte, C. & Ree, M. Water diffusion and sorption in films of high-performance poly(4,4'- oxydiphenylene pyromellitimide): effects of humidity, imidization history and film thickness. *Polymer* 36, 1663 – 1672 (1995). URL http://www.sciencedirect.com/science/article/ pii/003238619599012J. DOI https://doi.org/10.1016/0032-3861(95)99012-J.
2. Sacher, E. & Susko, J. R. Water permeation of polymer films. i. polyimide. *Journal of Applied Polymer Science* 23, 2355–2364 (1979). URL http://dx.doi.org/10.1002/app.1979. 070230813. DOI 10.1002/app.1979.070230813.
